# Exploring the toxicological network in diabetic microvascular disease: a commentary

**DOI:** 10.1097/JS9.0000000000004683

**Published:** 2026-01-13

**Authors:** Guosheng Lin, Fenglian Yu, Fangfeng Lin

**Affiliations:** a Department of Oncology, The Third Affiliated People’s Hospital of Fujian University of Traditional Chinese Medicine, Fuzhou, China; b Fujian University of Traditional Chinese Medicine Fuzhou, China; c Department of Endocrinology, The Third Affiliated People’s Hospital of Fujian University of Traditional Chinese Medicine, Fuzhou, China

*Dear Editor,*

We read with great interest the article by Song *et al*, entitled “Exploring the toxicological network in diabetic microvascular disease”^[1]^. The authors should be congratulated for systematically integrating network toxicology, protein–protein interaction (PPI) analysis, molecular docking, and multi-omics validation to investigate how endocrine-disrupting chemicals (EDCs) may contribute to diabetic kidney disease (DKD), diabetic retinopathy (DR), and distal symmetrical polyneuropathy (DSPN). Their work provides valuable hypotheses regarding shared hub targets, such as EGFR, ALB, MYC, ESR1, HSP90AA1, BCL2, and CD4, and offers a useful starting point for understanding EDC-related microvascular toxicity. However, we would like to raise several methodological points concerning the construction and visualization of the PPI networks and the interpretation of the molecular docking results. This commentary complies with the “2025 TITAN Guidelines” for statements and use in managing artificial intelligence^[2]^. No artificial intelligence tools were used during the writing of this work.

First, Song *et al* predicted EDC-related targets using SwissTargetPrediction and SEA, collected diabetic microvascular disease–related genes from CTD, GeneCards, and OMIM, and then intersected these datasets to obtain common targets for DKD (843), DR (474), and DSPN (623). These were imported into STRING and visualized in Cytoscape, followed by MCODE and CytoHubba analyses to identify core modules and the top 10 hub genes for each complication^[1]^. While this workflow is rigorous, the PPI figures (STRING output and the corresponding Cytoscape networks in Figures 1–5) are visually very dense, with hundreds of nodes and numerous edges displayed simultaneously. As a result, it is difficult for readers to intuitively recognize key modules, interaction patterns, or cross-disease similarities from the network images themselves; most mechanistic information must instead be inferred from the text and enrichment plots.

To enhance interpretability, future work could consider tightening the interaction confidence threshold in STRING and presenting only the most informative subnetworks (for example, MCODE-defined core clusters or hub-centered subnetworks) in the main figures while relegating the full PPI networks to supplementary material. Such adjustments would not change the underlying analyses but could make the graphical results more concise and more helpful for readers attempting to understand the proposed toxicological network. This strategy has been widely used in bioinformatics studies that apply MCODE/CytoHubba to extract and visualize the most biologically relevant protein complexes and hub genes from large PPI networks^[3]^.

Second, the authors used Discovery Studio 2019 and the CDOCKER algorithm to perform molecular docking between 12 representative EDCs and the top five hub targets identified in each PPI network. Receptor structures were prepared from the Protein Data Bank, binding pockets were predicted, and CDOCKER interaction energies were calculated; heatmaps and representative complexes were then shown for DKD, DR, and DSPN. Docking provides a reasonable first step for assessing potential ligand–target affinity. However, because both EDCs and protein binding pockets are conformationally flexible, single-frame docking scores may not fully capture the stability and realism of these interactions. The current conclusions that “EDCs have strong binding affinities with the top five hub targets” are therefore still somewhat preliminary.

To further substantiate these structure-based findings, future work could consider complementing docking with all-atom molecular dynamics (MD) simulations and post-docking free-energy estimation (e.g., MM/PBSA or MM/GBSA), which are widely used end-state methods for estimating protein–ligand binding free energies and can provide a more reliable ranking of candidate toxicants^[4,5]^. Even relatively short MD simulations, combined with an analysis of root-mean-square deviation/fluctuation, hydrogen-bond occupancy, and binding free energy, can provide more robust evidence regarding the stability and plausibility of specific EDC–target complexes.

In summary, Song *et al* have made an important contribution by mapping a comprehensive toxicological network linking EDCs to diabetic microvascular complications and by highlighting several shared hub targets across DKD, DR, and DSPN. Our comments focus on technical refinements that, in our view, could further improve the interpretability of the PPI networks and strengthen the structural evidence supporting ligand–target interactions. In addition, emerging machine learning approaches for multi-omics integration and toxicological target prioritization may further enhance such network-based frameworks in future studies^[6,7]^. We hope that these suggestions will be helpful for future work and will stimulate further discussion on methodological standards in network toxicology for endocrine disruptors.

## Data Availability

Not applicable.
